# Supplementary material for: Beauty of symmetry ‐ The impact of logo symmetry on perceived product quality
Source: PLoS One. 2025 Jan 28;20(1):e0317229. doi: 10.1371/journal.pone.0317229 (PMC11774398; doi:10.1371/journal.pone.0317229)
Supplement: S1 File — (DOCX) [file pone.0317229.s001.docx]

# Beauty of symmetry - The impact of logo symmetry on perceived product quality

Supporting Information

# Appendix 1: Minimal data set

| Study 1 | Perceived quality | | | Familiarity | | | Liking of product | | |
| --- | --- | --- | --- | --- | --- | --- | --- | --- | --- |
| Group | N | Mean | SD | N | Mean | SD | N | Mean | SD |
| Symmetrical Logo | 66 | 4.92 | 1.69 | 66 | 4.06 | 1.86 | 66 | 4.48 | 1.52 |
| Asymmetrical Logo | 66 | 4.19 | 1.33 | 66 | 4.12 | 1.88 | 66 | 4.24 | 1.62 |

| Pretest of Study 1 | Manipulation check | | | Liking of Logo | | | Aesthetic perception | | |
| --- | --- | --- | --- | --- | --- | --- | --- | --- | --- |
| Group | N | Mean | SD | N | Mean | SD | N | Mean | SD |
| Symmetrical Logo | 33 | 5.15 | 1.7 | 33 | 4.48 | 1.37 | 33 | 4.15 | 1.94 |
| Asymmetrical Logo | 33 | 2.06 | 1.22 | 33 | 4.45 | 1.73 | 33 | 4.67 | 1.87 |

| Study 2 | Perceived quality | | | Perceived stability | | | Perceived naturalness | | |
| --- | --- | --- | --- | --- | --- | --- | --- | --- | --- |
| Group | N | Mean | SD | N | Mean | SD | N | Mean | SD |
| Symmetrical Logo | 84 | 4.63 | 1.75 | 84 | 4.36 | 1.8 | 84 | 3.89 | 1.84 |
| Asymmetrical Logo | 84 | 3.71 | 1.56 | 84 | 3.71 | 1.69 | 84 | 4.02 | 2.18 |

| Study 2 | Manipulation check | | | Familiarity | | | Liking of product | | |
| --- | --- | --- | --- | --- | --- | --- | --- | --- | --- |
| Group | N | Mean | SD | N | Mean | SD | N | Mean | SD |
| Symmetrical Logo | 84 | 5.27 | 1.9 | 84 | 4.39 | 1.5 | 84 | 4.29 | 1.57 |
| Asymmetrical Logo | 84 | 2.3 | 1.27 | 84 | 4.51 | 1.32 | 84 | 4.24 | 1.63 |

| Study 3 | Perceived quality | | | Manipulation check of cognitive load | | |
| --- | --- | --- | --- | --- | --- | --- |
| Group | N | Mean | SD | N | Mean | SD |
| High cognitive load and Symmetrical Logo | 49 | 3.73 | 1.32 | 49 | 4.98 | 1.23 |
| High cognitive load and Asymmetrical Logo | 50 | 4.02 | 1.5 | 50 | 4.9 | 1.46 |
| Low cognitive load and Symmetrical Logo | 50 | 4.83 | 1.06 | 50 | 3.24 | 1.46 |
| Low cognitive load and Asymmetrical Logo | 50 | 4.09 | 1.37 | 50 | 3.46 | 1.51 |

| Pretest of Study 3 | Manipulation check | | | Liking of Logo | | | Aesthetic perception | | |
| --- | --- | --- | --- | --- | --- | --- | --- | --- | --- |
| Group | N | Mean | SD | N | Mean | SD | N | Mean | SD |
| Symmetrical Logo | 31 | 4.87 | 2.05 | 31 | 4.52 | 1.75 | 31 | 4.58 | 1.59 |
| Asymmetrical Logo | 30 | 2.9 | 1.37 | 30 | 4.13 | 1.72 | 30 | 4.57 | 1.3 |
