# Supplementary material for: Beauty of symmetry ‐ The impact of logo symmetry on perceived product quality
Source: PLoS One. 2025 Jan 28;20(1):e0317229. doi: 10.1371/journal.pone.0317229 (PMC11774398; doi:10.1371/journal.pone.0317229)
Supplement: Attachment — Submitted filename: Responses to reviewers.docx [file pone.0317229.s008.docx]

**Responses to the Editor and Reviewers’ Comments**

**Manuscript ID PONE-D-24-32602**

**Dear Editor and Reviewers:**

Thank you for your letter and for the reviewers’ comments concerning our manuscript entitled “Beauty of Symmetry - The Impact of Logo Symmetry on Perceived Product Quality” (ID: PONE-D-24-32602). These comments were very helpful for revising and improving our paper as well as for guiding our research. We have studied the comments carefully and have made corrections that we hope are satisfactory. The revised text is marked with **different colour** in the paper. We have indicated the location of the revisions, including the line and page numbers, to make finding the changed/added content easier in the manuscript. Overall, We have enhanced the introduction and conclusion sections and added more evidence to support our research. Furthermore, we have revised the formatting of the entire paper to eliminate grammatical errors.

According to the editor's guidance, we have replied to each reviewer's comments in a 3-column table.

**Responses to Additional Editor**

| **Comment** | **Response to the comment** | **Changed/Added in the manuscript** |
| --- | --- | --- |
| **# Additional Editor Comments**  This study can potentially contribute to the literature. However, it should be made more scientific with some corrections. | I appreciate your positive and constructive comments regarding our manuscript. Your detailed comments have provided us with clear directions for modification.  Below, I respond to each of your comments. We have made every effort to revise and improve the manuscript according to your comments. I hope that our responses and the revised manuscript meet your expectations. |  |
| **#comment 1:**  First of all, the introduction seems short. The introduction should be expanded and the main contribution of the study should be stated. | Thank you for the feedback. I have expanded and refined the Introduction, addressing the research gaps pertinent to this study and highlighting our contribution to marketing research. | I have added contents to the “Introduction”. The details are as follows :  “*One of the key factors in logo design is symmetry. Symmetrical designs are often perceived as more attractive, balanced, and credible (Cárdenas and Harris, 2006). This preference for symmetry is rooted in cognitive processing; symmetrical visuals are easier to process, leading to a perception of harmony and stability (Bertamini and Makin, 2014). Therefore, exploring how logo symmetry influences consumers' perception of product quality is of both theoretical and practical importance, as it can help businesses create logos that foster positive consumer evaluations and brand loyalty (Bettels and Wiedmann, 2019).*”  “*This study seeks to address this gap by examining how logo symmetry influences consumers' perception of product quality.*”  “*Our findings contribute to the literature on logo design and visual perception in several ways. First, this study demonstrate that logo symmetry directly impacts consumer perception of product attributes, providing insights into how visual elements influence consumer evaluations beyond mere aesthetics. Second, this study introduce perceived stability as a mediating factor, expanding the understanding of how logo design affects consumer behavior. Finally, this study highlight the role of cognitive load in moderating the effect of logo symmetry, suggesting that the effectiveness of visual design elements can vary depending on the cognitive context of consumers.*  *From a practical perspective, this study provides actionable insights for marketers and designers. Companies can enhance consumers' perceived quality of their products by adopting symmetrical logos, especially for products that need to convey stability and trust (Bettels and Wiedmann, 2019). Furthermore, marketing strategies should consider the cognitive load of the target audience - symmetrical logos are more effective when consumers are not overwhelmed by competing stimuli. This study also opens avenues for future studies to explore the impact of other visual elements, such as color and shape complexity, on consumer behavior, particularly across different cultural contexts. In summary, by investigating the impact of logo symmetry on perceived product quality and the mechanisms behind this effect, this study aims to advance the understanding of logo design's role in consumer perception. These findings are not only theoretically significant but also offer practical guidance for effective marketing and branding strategies in today’s visually driven marketplace.*”  (Section “Introduction” on Line 27 - 33, Line 44 - 45, Line 51 - 69) |
| **#comment 2:**  The first person singular we and i should not be used in the study. There should be expressions such as this study or in this study instead of we and our. | Thank you for your corrections regarding the wording in this study. I now realize that first-person singular should be avoided in describing this research, and I have revised all expressions that were inconsistent with this convention. | I have changed “I”, “We”, and “Our” into “This Study” |
| **#comment 3:**  The conclusion of the study should be written in more detail with previous literature. The differences or similarities between the previous literature and the current literature are emphasized. More policy recommendations should be given. limitations and future directions should be stated. | Thank you for your constructive suggestions regarding the study’s limitations in the conclusion. These insights are invaluable for strengthening the manuscript. Based on the recommendations, I have expanded the conclusion section to include comparisons with existing literature and additional policy suggestions. Furthermore, we have considered other potential research directions, thereby increasing this study’s relevance as a reference for future research. | I have added contents to the manuscript. The details are as follows :  “*Finally, this study also emphasizes both similarities and differences with previous literature. While prior studies (e.g., Bettels and Wiedmann, 2019; Krey and Rossi, 2018) highlighted the aesthetic and symbolic role of symmetry, this research extends these findings by showing a direct impact on perceived product quality through perceived stability. Additionally, while the concept of perceived stability has been discussed in other contexts such as price (Turnovsky et al., 1980) and round numbers (Pena‐Marin and Bhargave, 2016), this work uniquely applies it to visual design, thereby broadening the scope of perceived stability in consumer behavior research.*”  (Section “Theoretic Contribution” on Line 429 - 435)  “*Finally, based on the findings of this study, policymakers should support initiatives aimed at promoting effective visual branding, especially for businesses involved in consumer goods. Encouraging companies to adopt symmetrical logo designs can enhance consumer perceptions of product quality and stability, thereby contributing to overall market efficiency. Additionally, regulatory bodies could provide guidelines on marketing practices that minimize cognitive load for consumers, ensuring that important product information is effectively communicated (Drichoutis, 2017). This could involve setting standards for advertising in environments with high cognitive complexity, such as social media platforms.*”  (Section “Practical Contribution” on Line 465 - 472)  “*Another limitation is the use of a controlled setting that may not fully capture the nuances of consumer decision-making in natural environments. Future research could employ field studies or real-world experiments to examine how logo symmetry impacts perceived quality in different consumer settings, such as retail stores or digital marketplaces. Moreover, future research could explore other visual elements, such as complexity (Mahmood et al., 2019), curvature (Palumbo and Bertamini, 2016), and dynamism (Cian et al., 2014), and their combined effects on consumer perception. The cross-cultural applicability of these findings is another area for further investigation, as visual preferences and cognitive processing may vary significantly across different cultural contexts.*”  (Section “Limitation and Future Research” on Line 484 - 492) |
| **#comment 4:**  Also, all text must be rearranged for stitching. | Thank you for your valuable suggestions on this manuscript. I have revised it to align with the formatting and layout guidelines required by PLOS ONE, ensuring full compliance with their standards. Additionally, I have expanded the research content in response to your earlier recommendations. | Please refer to the manuscript for detailed revisions, which have been highlighted in different color for easy identification. |
| **#comment 5:**  Hypothesis setups should be revised. Hypotheses should be established with more scientific and clear sentences. | Thank you for your suggestions on the hypothesis setups section. Based on your feedback, I have refined the hypothesis development section by adopting more rigorous academic language and adding real-world examples to improve the clarity and accessibility of this study. | I have changed contents to the section Hypothesis Development. The details are as follows :  “*Symmetrical logos are fundamental in enhancing visual stability, making them inherently more appealing and leading to a more positive perception among consumers. Empirical studies have consistently demonstrated that symmetrical designs are perceived as more aesthetically pleasing, thereby significantly influencing consumer interactions with logos and brand elements (Bajaj & Bond, 2017). The concept of symmetry extends beyond mere visual attractiveness; it conveys attributes such as balance, order, and stability, which collectively contribute to a positive perception of a brand's intrinsic values. This preference for symmetry indicates that symmetrical logos evoke a sense of coherence, elegance, and high quality, which ultimately leads to more favorable consumer attitudes and a deeper emotional connection with the brand (Creusen et al., 2010). For example, the logos of brand like Toyota is quintessential example of symmetry that convey these positive associations, contributing to their strong brand identities. Furthermore, the consistent use of symmetrical logos fosters brand identity, reinforcing consumer trust and loyalty by evoking a sense of familiarity and stability (Coelho et al., 2019). In addition, symmetry is frequently associated with permanence and reliability—key attributes that are crucial for brands seeking to establish long-term relationships with consumers, such as in the case of the symmetrical logo of McDonald's, which conveys reliability and endurance (Pena‐Marin & Bhargave, 2015).*  *A substantial body of literature has demonstrated that stable visual elements significantly enhance consumer perceptions of product quality, durability, and shelf life. For instance, Coelho et al. (2019) illustrate how stable shapes contribute not only to a positive brand image but also to an enhanced perception of product quality, while Pena‐Marin and Bhargave (2019) argue that products perceived as having stable attributes are evaluated more favorably by consumers. This can be observed in the packaging of high-end perfumes, which often use sturdy, symmetrical bottles to convey a sense of premium quality and stability. Similarly, Yan et al. (2013) noted that smaller, meticulously designed products are often seen as more challenging to manufacture, thereby implying higher quality. Applying this concept to stable shapes, products that visually signal stability—such as the iconic Coca-Cola glass bottle—are often perceived as having higher quality. Mitra & Golder (2006) highlight the impact of objective quality on consumer perceptions, emphasizing that stability in design fosters consumer trust and loyalty (Leischnig & Enke, 2011). For example, the durable and stable design of Tupperware products conveys longevity and reliability, which positively influences consumer perceptions of quality and durability. In conclusion, products with stable visual elements are more likely to enhance consumer perceptions of quality, significantly shaping evaluations of product longevity and fostering a positive brand image.* ”  (Section “Hypothesis Development - Logo Symmetry and Perceived Quality” on Line 130 - 163)  “*Cognitive load refers to the mental effort required to process information in working memory. When cognitive load is low, individuals have greater cognitive resources available to engage with and appreciate complex stimuli, such as the symmetry of logos (Sweller, 1988). Conversely, under conditions of high cognitive load, these cognitive resources are largely occupied, which can hinder the ability to process intricate visual details. For example, consumers shopping online during a relaxed browsing session (low cognitive load) may be more capable of appreciating a brand logo's symmetrical design than when multitasking or in a rushed environment (high cognitive load). Research suggests that high logo symmetry is associated with higher perceived quality under low cognitive load, whereas this effect diminishes under high cognitive load. This is supported by findings that cognitive load influences visual perception and attention mechanisms. Lavie et al. (2004) and Konstantinou and Lavie (2013) demonstrated that individuals have greater capacity to process visual details, such as symmetry, under low cognitive load conditions, leading to enhanced perception and evaluation of these stimuli. Moreover, empirical studies by Saiphoo and Want (2018) and Derpsch et al. (2021) indicate that cognitive load plays a significant role in shaping the evaluation of visual information, further supporting the notion that symmetry is more effectively appreciated when cognitive resources are not heavily taxed. In a practical context, this suggests that brands aiming to convey a sense of quality through logo design should consider the cognitive state of their target audience. For example, advertising campaigns during times when consumers are likely to be relaxed (e.g., during weekends or leisure activities) may enhance the positive effect of symmetrical logos on perceived quality.*”  (Section “Hypothesis Development - Moderator role of cognitive load” on Line 171 - 190) |

**Responses to Reviewer #1**

| **Comment** | **Response to the comment** | **Changed/Added in the manuscript** |
| --- | --- | --- |
| **#overall:**  This article investigates the effect of logo symmetry on perceived product quality, and as a scientific reviewer, it can be evaluated from the following perspectives | Thank you for your constructive feedback on this study. I have revised the entire manuscript according to your suggestions. |  |
| **#comment 1:**  Literature Review and Theoretical Foundation: The article provides a detailed literature review supporting the effect of logo symmetry on perceived product quality. However, it would benefit from referencing more recent studies and discussing the influence of other logo design elements in greater depth beyond symmetry. | I also appreciate your acknowledgment of the literature review and your guidance on addressing certain limitations. In the “Limitations and Future Research” section, I have added a summary of current findings on logo symmetry and discussed related research gaps. | I have added contents to the manuscript. The details are as follows :  “*Moreover, future research could explore other visual elements, such as complexity (Mahmood et al., 2019), curvature (Palumbo and Bertamini, 2016), and dynamism (Cian et al., 2014), and their combined effects on consumer perception. The cross-cultural applicability of these findings is another area for further investigation, as visual preferences and cognitive processing may vary significantly across different cultural contexts.*”  (Section “Limitation and Future Research” on Line 484 - 492) |
| **#comment 2:**  Hypothesis Development: The hypotheses are clearly stated, yet the hypothesis that “symmetrical logos increase perceived quality” could be grounded in a more concrete theoretical framework. Strengthening theoretical explanations, particularly regarding symmetry’s impact on cognitive processing alongside its aesthetic value, would be beneficial. | Thank you for your suggestions on the hypothesis development section of this study. Based on your feedback, I have refined the hypotheses to strengthen their logical clarity and improve readability. | I have changed contents to the section Hypothesis Development. The details are as follows :  “*Symmetrical logos are fundamental in enhancing visual stability, making them inherently more appealing and leading to a more positive perception among consumers. Empirical studies have consistently demonstrated that symmetrical designs are perceived as more aesthetically pleasing, thereby significantly influencing consumer interactions with logos and brand elements (Bajaj & Bond, 2017). The concept of symmetry extends beyond mere visual attractiveness; it conveys attributes such as balance, order, and stability, which collectively contribute to a positive perception of a brand's intrinsic values. This preference for symmetry indicates that symmetrical logos evoke a sense of coherence, elegance, and high quality, which ultimately leads to more favorable consumer attitudes and a deeper emotional connection with the brand (Creusen et al., 2010). For example, the logos of brand like Toyota is quintessential example of symmetry that convey these positive associations, contributing to their strong brand identities. Furthermore, the consistent use of symmetrical logos fosters brand identity, reinforcing consumer trust and loyalty by evoking a sense of familiarity and stability (Coelho et al., 2019). In addition, symmetry is frequently associated with permanence and reliability—key attributes that are crucial for brands seeking to establish long-term relationships with consumers, such as in the case of the symmetrical logo of McDonald's, which conveys reliability and endurance (Pena‐Marin & Bhargave, 2015).*  *A substantial body of literature has demonstrated that stable visual elements significantly enhance consumer perceptions of product quality, durability, and shelf life. For instance, Coelho et al. (2019) illustrate how stable shapes contribute not only to a positive brand image but also to an enhanced perception of product quality, while Pena‐Marin and Bhargave (2019) argue that products perceived as having stable attributes are evaluated more favorably by consumers. This can be observed in the packaging of high-end perfumes, which often use sturdy, symmetrical bottles to convey a sense of premium quality and stability. Similarly, Yan et al. (2013) noted that smaller, meticulously designed products are often seen as more challenging to manufacture, thereby implying higher quality. Applying this concept to stable shapes, products that visually signal stability—such as the iconic Coca-Cola glass bottle—are often perceived as having higher quality. Mitra & Golder (2006) highlight the impact of objective quality on consumer perceptions, emphasizing that stability in design fosters consumer trust and loyalty (Leischnig & Enke, 2011). For example, the durable and stable design of Tupperware products conveys longevity and reliability, which positively influences consumer perceptions of quality and durability. In conclusion, products with stable visual elements are more likely to enhance consumer perceptions of quality, significantly shaping evaluations of product longevity and fostering a positive brand image.* ”  (Section “Hypothesis Development - Logo Symmetry and Perceived Quality” on Line 130 - 163) |
| **#comment 3:**  Methodology and Experimental Design: The three experiments used in the study offer an appropriate methodological approach to test the hypotheses. However, expanding the participant pool to increase the generalizability of the findings would be recommended. Additionally, the exclusive use of online experiments may overlook the complex influences present in real-world environments. | Thank you for your valuable suggestions regarding the experimental section of this study. Your recommendation to expand the participant pool is insightful, as a larger and more diverse sample could indeed improve the generalizability of our findings. To broaden the applicability of this study, we incorporated a variety of stimuli, which serves as an initial but constrained approach to expanding the study’s scope within the field of marketing research. Additionally, we have included an additional limitation in the limitations section for further reference. | I have added contents to the manuscript. The details are as follows :  “*Another limitation is the use of a controlled setting that may not fully capture the nuances of consumer decision-making in natural environments. Future research could employ field studies or real-world experiments to examine how logo symmetry impacts perceived quality in different consumer settings, such as retail stores or digital marketplaces.* ”  (Section “Limitation and Future Research” on Line 484 - 492) |
| **#comment 4:**  Presentation of Findings: The findings are clearly presented, but further interpretation of the statistical analyses could be useful. In particular, detailing the role of cognitive load would help readers better understand this factor’s influence. | Thank you for your suggestions on this study. I appreciate your acknowledgment of the clarity in presenting the findings. I will work on strengthening the interpretation of the statistical analyses, particularly through a more in-depth discussion of the role of cognitive load. Additionally, I have included a more detailed explanation of cognitive load in the hypothesis development section to enhance the accessibility of the theoretical framework. | I have added contents to the manuscript. The details are as follows :  “*Cognitive load refers to the mental effort required to process information in working memory. When cognitive load is low, individuals have greater cognitive resources available to engage with and appreciate complex stimuli, such as the symmetry of logos (Sweller, 1988). Conversely, under conditions of high cognitive load, these cognitive resources are largely occupied, which can hinder the ability to process intricate visual details. For example, consumers shopping online during a relaxed browsing session (low cognitive load) may be more capable of appreciating a brand logo's symmetrical design than when multitasking or in a rushed environment (high cognitive load). Research suggests that high logo symmetry is associated with higher perceived quality under low cognitive load, whereas this effect diminishes under high cognitive load. This is supported by findings that cognitive load influences visual perception and attention mechanisms. Lavie et al. (2004) and Konstantinou and Lavie (2013) demonstrated that individuals have greater capacity to process visual details, such as symmetry, under low cognitive load conditions, leading to enhanced perception and evaluation of these stimuli. Moreover, empirical studies by Saiphoo and Want (2018) and Derpsch et al. (2021) indicate that cognitive load plays a significant role in shaping the evaluation of visual information, further supporting the notion that symmetry is more effectively appreciated when cognitive resources are not heavily taxed. In a practical context, this suggests that brands aiming to convey a sense of quality through logo design should consider the cognitive state of their target audience. For example, advertising campaigns during times when consumers are likely to be relaxed (e.g., during weekends or leisure activities) may enhance the positive effect of symmetrical logos on perceived quality.*”  (Section “Hypothesis Development - Moderator Role of Cognitive Load” on Line 171 - 190) |
| **#comment 5:**  Theoretical and Practical Contributions: The article articulates its theoretical contributions well, though it would be beneficial to support the practical recommendations with specific application examples. This would provide a more concrete guide for businesses regarding logo design considerations. | Thank you for your suggestions on improving the theoretical and practical contributions. After carefully reviewing your feedback, I have included additional elaboration in the final “Theoretical and Practical Contributions” section. | I have added contents to the manuscript. The details are as follows :  “*Finally, this study also emphasizes both similarities and differences with previous literature. While prior studies (e.g., Bettels and Wiedmann, 2019; Krey and Rossi, 2018) highlighted the aesthetic and symbolic role of symmetry, this research extends these findings by showing a direct impact on perceived product quality through perceived stability. Additionally, while the concept of perceived stability has been discussed in other contexts such as price (Turnovsky et al., 1980) and round numbers (Pena‐Marin and Bhargave, 2016), this work uniquely applies it to visual design, thereby broadening the scope of perceived stability in consumer behavior research.*”  (Section “Theoretic Contribution” on Line 429 - 435)  “*Finally, based on the findings of this study, policymakers should support initiatives aimed at promoting effective visual branding, especially for businesses involved in consumer goods. Encouraging companies to adopt symmetrical logo designs can enhance consumer perceptions of product quality and stability, thereby contributing to overall market efficiency. Additionally, regulatory bodies could provide guidelines on marketing practices that minimize cognitive load for consumers, ensuring that important product information is effectively communicated (Drichoutis, 2017). This could involve setting standards for advertising in environments with high cognitive complexity, such as social media platforms.*”  (Section “Practical Contribution” on Line 465 - 472) |
| **#comment 6:**  Conclusion and Limitations: While the article summarizes its research findings and offers suggestions for future studies, emphasizing the limitations that might be encountered in real-world applications could further enhance the reliability of the study. | We appreciate your insightful feedback on our article's conclusion and limitations section. Emphasizing the real-world limitations of our findings is a valuable suggestion. Accordingly, we have highlighted the study’s limitations regarding real-world applications in the limitations section, with the goal of offering clearer directions for future research. | I have added contents to the manuscript. The details are as follows :  “*Another limitation is the use of a controlled setting that may not fully capture the nuances of consumer decision-making in natural environments. Future research could employ field studies or real-world experiments to examine how logo symmetry impacts perceived quality in different consumer settings, such as retail stores or digital marketplaces. Moreover, future research could explore other visual elements, such as complexity (Mahmood et al., 2019), curvature (Palumbo and Bertamini, 2016), and dynamism (Cian et al., 2014), and their combined effects on consumer perception. The cross-cultural applicability of these findings is another area for further investigation, as visual preferences and cognitive processing may vary significantly across different cultural contexts.*”  (Section “Limitation and Future Research” on Line 484 - 492) |
| **#comment 7:**  In conclusion, the article has a generally strong structure; however, it could be enriched by adding more depth in areas such as the literature review, theoretical framework, methodological details, and practical contributions, making it scientifically more robust. | Thank you for your thoughtful and constructive feedback. I appreciate your recognition of the article's overall structure. I understand the importance of adding depth in the areas you've highlighted. Enhancing the literature review, theoretical framework, and methodological details, as well as clarifying practical contributions, would indeed strengthen the scientific rigor and applicability of our work. I will carefully consider these suggestions in our revisions to ensure a more robust and comprehensive final paper. |  |

**Responses to Reviewer #2**

| **Comment** | **Response to the comment** | **Changed/Added in the manuscript** |
| --- | --- | --- |
| **#comment 1:**  A few explanatory sentences about the symmetry effect can be added to the introduction. | Thank you for your feedback on the introduction section of this manuscript. The introduction did indeed previously lack a description of the concept of the symmetry effect. Based on your suggestions, I have now included an explanation of this concept in the introduction. | I have added contents to the manuscript. The details are as follows :  “*One of the key factors in logo design is symmetry. Symmetrical designs are often perceived as more attractive, balanced, and credible (Cárdenas and Harris, 2006). This preference for symmetry is rooted in cognitive processing; symmetrical visuals are easier to process, leading to a perception of harmony and stability (Bertamini and Makin, 2014). Therefore, exploring how logo symmetry influences consumers' perception of product quality is of both theoretical and practical importance, as it can help businesses create logos that foster positive consumer evaluations and brand loyalty (Bettels and Wiedmann, 2019).*”  (Section “Introduction” on Line 27 - 33, Line 44 - 45, Line 51 - 69) |
| **#comment 2:**  The discussion sections of the study should be expanded in a more explanatory manner. | Thank you for your revision suggestions for each section of this manuscript. In the discussion section of each study, I have added expanded summaries of the research content and outlined the limitations of each study, as well as discussed their relationship to findings in other sections of the manuscript. | I have added contexts to the discussion of each study. The details are as follows:  “*While Study 1 directly verified the main effect of logo symmetry on perceived product quality, it did not investigate the underlying mechanisms of this effect, leaving the explanatory scope incomplete. Therefore, Study 2 aimed to further explore the potential mediating processes. This study hypothesize that the positive influence of logo symmetry on perceived product quality is mediated by perceived stability; specifically, a symmetrical logo may lead consumers to form a higher perception of stability, which in turn enhances perceived product quality.*”  (Section “Discussion of Study 1” on Line 258 - 264)  “*Drawing on the results from Studies 1 and 2, this research confirmed both the positive impact of logo symmetry on perceived quality and the underlying mechanisms of this effect. In Study 3, this study investigated the moderating effect of this influence to strengthen the relevance of this findings within practical marketing contexts.*”  (Section “Discussion of Study 2” on Line 326 - 329)  “In addition, Study 3 employed stimuli different from those in Studies 1 and 2 to further assess the generalizability of logo symmetry’s effect on perceived quality across various product categories. The three studies tested the research hypotheses across diverse stimulus conditions. Overall, empirical support was found for Hypotheses 1, 2, and 3.”  (Section “Discussion of Study 3” on Line 387 - 391) |
| **#comment 3:**  The general discussion section should also be expanded. | Thank you for your suggestions on the general discussion section of this manuscript. Following your advice, I have added comparisons between this study and prior research in the “Theoretical Contribution”, “Practical Contribution,” and “Limitations and Future Research” sections, along with recommendations for policymakers and additional potential avenues for future research. | I have added contents to the “Generally Discussion”. The details are as follows :  “*Finally, this study also emphasizes both similarities and differences with previous literature. While prior studies (e.g., Bettels and Wiedmann, 2019; Krey and Rossi, 2018) highlighted the aesthetic and symbolic role of symmetry, this research extends these findings by showing a direct impact on perceived product quality through perceived stability. Additionally, while the concept of perceived stability has been discussed in other contexts such as price (Turnovsky et al., 1980) and round numbers (Pena‐Marin and Bhargave, 2016), this work uniquely applies it to visual design, thereby broadening the scope of perceived stability in consumer behavior research.*”  (Section “Theoretic Contribution” on Line 428 - 434)  “*Finally, based on the findings of this study, policymakers should support initiatives aimed at promoting effective visual branding, especially for businesses involved in consumer goods. Encouraging companies to adopt symmetrical logo designs can enhance consumer perceptions of product quality and stability, thereby contributing to overall market efficiency. Additionally, regulatory bodies could provide guidelines on marketing practices that minimize cognitive load for consumers, ensuring that important product information is effectively communicated (Drichoutis, 2017). This could involve setting standards for advertising in environments with high cognitive complexity, such as social media platforms.*”  (Section “Practical Contribution” on Line 464 - 471)  “*Another limitation is the use of a controlled setting that may not fully capture the nuances of consumer decision-making in natural environments. Future research could employ field studies or real-world experiments to examine how logo symmetry impacts perceived quality in different consumer settings, such as retail stores or digital marketplaces. Moreover, future research could explore other visual elements, such as complexity (Mahmood et al., 2019), curvature (Palumbo and Bertamini, 2016), and dynamism (Cian et al., 2014), and their combined effects on consumer perception. The cross-cultural applicability of these findings is another area for further investigation, as visual preferences and cognitive processing may vary significantly across different cultural contexts.*”  (Section “Limitation and Future Research” on Line 483 - 491) |
